# Hybrid Dissolving Microneedle-Mediated Delivery of Ibuprofen: Solubilization, Fabrication, and Characterization

**DOI:** 10.3390/ph16050677

**Published:** 2023-04-30

**Authors:** Talaya Hidayatullah, Fazli Nasir, Muzna Ali Khattak, Sadia Pervez, Waleed H. Almalki, Fawaz Alasmari, Gul e Maryam, Altaf ur Rahman, Arbab Tahir Ali

**Affiliations:** 1 Department of Pharmacy, University of Peshawar, Peshawar 25000, Pakistan; talayaarbab@uop.edu.pk (T.H.); muznaali@uop.edu.pk (M.A.K.); sadiapervez@uop.edu.pk (S.P.); altafrkhanutmanzai@gmail.com (A.u.R.); tahir.arbab@uop.edu.pk (A.T.A.); 2 Department of Pharmacy, CECOS University of IT and Emerging Sciences, Peshawar 25000, Pakistan; 3 Department of Pharmacology and Toxicology, College of Pharmacy, Umm Al-Qura University, Makkah P.O. Box 715, Saudi Arabia; whmalki@uqu.edu.sa; 4 Department of Pharmacology and Toxicology, College of Pharmacy, King Saud University, Riyadh 11451, Saudi Arabia; ffalasmari@ksu.edu.sa; 5 Department of Pharmacy, Qurtaba University of Science and Information Technology, Peshawar 25000, Pakistan; gulemaryam112@gmail.com

**Keywords:** ibuprofen, Soluplus, Poly vinyl alcohol, dissolving microneedles, pharmacokinetic parameters

## Abstract

Microneedles have recently emerged as a promising platform for delivering therapeutic agents by disrupting the skin, resulting in improved and high drug delivery via this route. Ibuprofen is widely used topically and orally for chronic pain conditions; to avoid untoward gastric effects, topical application is preferred over the oral route. This study aimed to enhance the solubility of the poorly water-soluble ibuprofen using Soluplus (SP) as a solubilizer and to fabricate dissolving microneedle patches of the drug. The fabricated patches were compared with marketed oral and topical formulations of ibuprofen. A 432-fold increase was observed in the solubility of the drug at 8% SP. The FTIR studies revealed that the drug and polymers were compatible. MNs were of uniform morphology and released the drug in a predictable manner. The in vivo analysis on healthy human volunteers revealed a Cmax of 28.7 µg/mL ± 0.5 with a T_max_ of 24 h and a MRT of 19.5 h, which was significantly higher than that observed for commercially available topical formulations. The prepared ibuprofen microneedles have higher bioavailability and MRT at a lower dose (165 µg) as compared to tablet and cream doses (200 mg).

## 1. Introduction

Transdermal delivery provides an attractive alternative to other routes due to multiple advantages, including pain-free, non-invasive, easy self-administration by the patient, and the first-pass effect. Molecules smaller in size can easily pass through the skin’s epithelial barrier, enabling effective and easy penetration [1,2]. The biological barrier of the stratum corneum layer of the skin serves as a major barrier for smaller and macromolecules to penetrate through this route [3]. A variety of strategies have been explored to overcome this barrier and increase percutaneous absorption [4]. These strategies include the use of various chemical enhancers, liquid-powder jet injections, iontophoresis, electroporation, microdermabrasion, ultrasound, thermal ablation, and microneedles (MNs) [5]. Microneedle arrays have been widely studied and explored to enhance drug delivery through the transdermal route. Microneedles (MN) are minimally invasive micron-size devices that are long enough to painlessly by-pass the skin’s stratum corneum (SC), which serves as the principal barrier to drugs that are applied topically [6]. MNs of various geometries are made of various materials, such as silicon, metals, glass, and polymers. MNs are classified into four types: solid MNs, coated MNs, dissolving MNs, and hollow MNs. Each type of MN has distinct advantages and disadvantages. Dissolving MNs have gained immense attention as they are advantageous over the other types due to their ability to encapsulate sensitive molecules; they offer high drug loading; they are made of water-soluble, biodegradable, relatively inexpensive polymers that dissolve upon insertion into the skin, leaving no sharp biohazardous waste after use; they involve easy manufacturing techniques; and they offer an economically suitable option for mass production [7].

Poly vinyl alcohol (PVA) is a biocompatible polymer that is widely used in the pharmaceutical industry due to its low cost, good aqueous solubility, easy handling, and availability in a number of grades. PVA has been previously found to produce MNs that are safe to be used in humans and possess sufficient mechanical strength [8,9].

**Figure 1:**
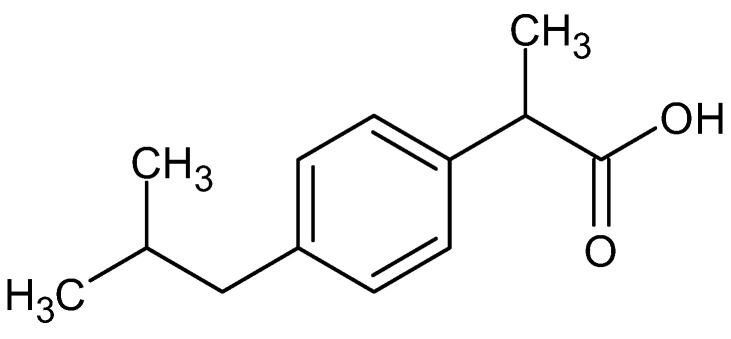
Molecular structure of ibuprofen.

In this study, ibuprofen has been used as a model drug for incorporation into dissolving microneedles. ibuprofen is a chiral non-steroidal anti-inflammatory drug of the 2-aryl propionic acid class with a molecular weight of 206.29 g/mol, and its chemical formula is C_13_H_18_O_2,_ as shown in Figure 1.

Different approaches have been used for transdermal ibuprofen delivery [10]. Ibuprofen, being a BCS class II drug, has poor aqueous solubility (less than 1 mg/mL) that results in low bioavailability and requires high doses and frequent administration as compared to its salt form, ibuprofen sodium, which has good aqueous dissolution, allowing faster absorption in the body [11,12,13]. Soluplus^®^ (BASF SE, Ludwigshafen, Germany) (molecular weight range of 90,000–140,000 g/mol) and a critical micellar concentration of 7.6 mg/L is capable of improving the aqueous solubility of poorly soluble compounds such as ibuprofen. It can self-assemble into micelles above the CMC due to its amphipathic nature [14]. Previously various strategies had been applied to formulate dissolving microneedle arrays, one such work was conducted by Donnelly et al., who tested various aqueous polymeric blends for the fabrication of DMN arrays loaded with ibuprofen sodium, various gel formulations of different polymers such as Poly lactic acid (PLA), Eudragit, Gantrez, Poly vinyl alcohol (PVA), Poly vinyl pyrrolidone (PVP) and other were used as matrix forming materials with high loading of ibuprofen sodium but most of these conventional polymer blends including PVA resulted in improperly formed microneedle array patches, the problems such as insoluble drug-polymer aggregates, distinct separation of drug and polymer, stamp molded microneedle arrays, or unformed brittle needles were observed [15]. Several other studies have also been carried out for the fabrication of ibuprofen sodium microneedles using non-ionic surfactants such as Tween 80, Pluronic F88, and Lutrol F108 to study the effect of strength incorporation by these surfactants [16]. Our study is focused on resolving the poor aqueous solubility of ibuprofen, achieving a stable polymer-drug formulation with PVA, and translating the use of PVA dissolving MNs patches in healthy human volunteers for transdermal delivery of ibuprofen. PVA is a biocompatible polymer that is commercially available. It has been used in the fabrication of microneedles due to its optimum physical strength [17,18], good aqueous solubility [19], and biocompatibility [20,21]. The poor aqueous solubility of ibuprofen was addressed by incorporating it in Soluplus^®^ micelles and using PVA as a matrix-forming material for loading into DMNs. This will significantly enhance ibuprofen bioavailability and patient compliance in arthritis patients who repeatedly need to take analgesics as part of their daily routine. To ensure effectiveness, the fabricated MNs will be compared with orally administered ibuprofen and topical ibuprofen-marketed formulations. The hypothesis of the study is to enhance the aqueous solubility of ibuprofen by using Soluplus^®^ as a solubility enhancer, which will result in higher bioavailability after administering the prepared dissolvable MNs.

## 2. Results and Discussions

### 2.1. Fourier Transform Infrared Spectroscopy (FTIR)

**Figure 2:**
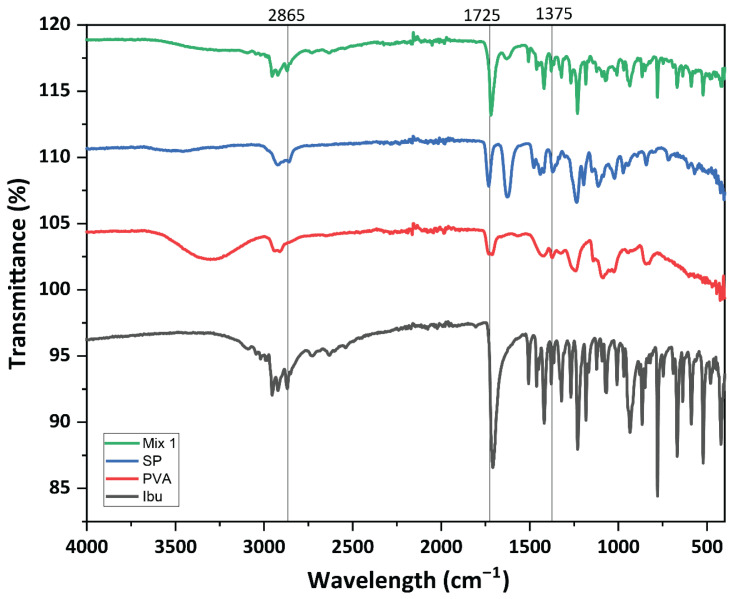
FTIR spectra of the drug (Ibu), polymer (PVA), Soluplus (SP) and their physical mixture (Mix 1).

FTIR spectra were obtained for ibuprofen, PVA, Soluplus^®^, and their physical mixtures. The % transmittance was measured in the spectral range of 4000 to 400 cm^−1^. The FTIR spectrum of pure ibuprofen showed well-defined and intense bands that are characteristic of ibuprofen at 1700 cm^−1^ (carbonyl stretching of the isopropionic acid group) and at 3000 cm^−1^ (hydroxyl stretching), as shown in Figure 2.

These peaks were retained in the physical mixture, showing no chemical interactions between the drug, PVA, and Soluplus. The C-H/O-H stretching of amine is characteristic between 3300 and 3400 cm^−1^ for PVA; this band is reduced in the physical mixture, showing physical interaction between the drug and the polymer.

### 2.2. Thermal Analysis (DSC/TGA)

**Figure 3:**
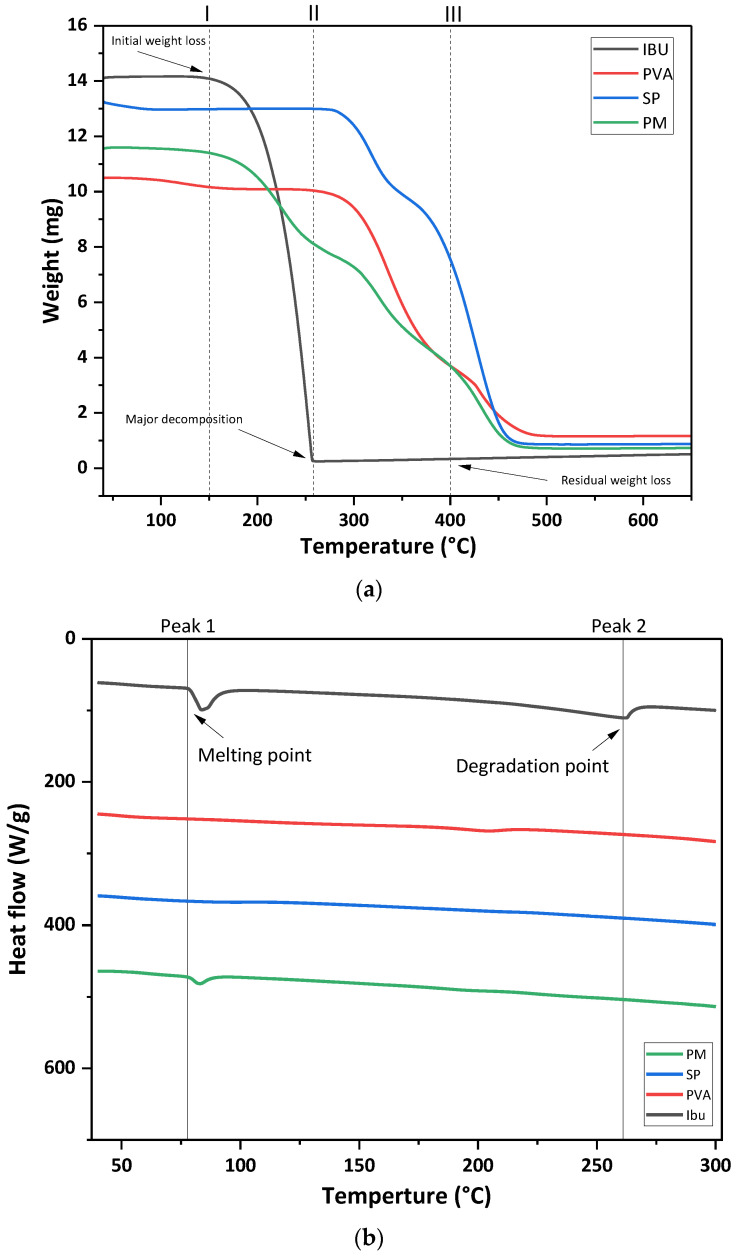
(**a**) TGA curves of ibuprofen, PVA, SP and their physical mixture (**b**) DSC thermogram of ibuprofen, polymers, and their physical mixture.

Differential scanning calorimetry (DSC) and thermal gravimetric analysis (TGA) were performed for the pure drug, the excipients, and the physical mixtures. TGA analysis was performed to assess the thermal stability of ibuprofen and the other excipients. Ibuprofen was found to be thermally stable when heated up to 200 °C, while Soluplus (SP) remained stable until 300 °C, beyond which the polymer degradation initiated. The physical mixture of the drug, PVA, and SP retained thermal stability up to 210 °C, as shown in Figure 3a. The DSC curve of pure ibuprofen showed an endothermic peak at around 75 °C, indicating the melting point of the drug, with a second peak at around 250 °C showing its degradation. However, the physical mixture enhanced the thermal stability of the drug, with no drug degradation at 250 °C, as shown in Figure 3b.

### 2.3. Solubilization of Ibuprofen

**Figure 4:**
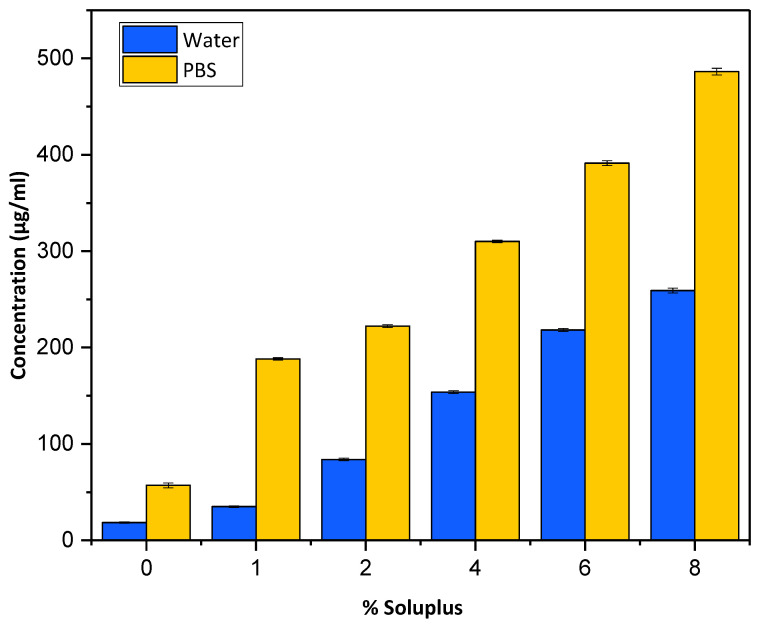
Solubility profile of ibuprofen in water and PBS with different concentrations of Soluplus.

The solubility of ibuprofen (n = 3) in distilled water and PBS at room temperature (~25 °C) was determined. Different Soluplus solutions were prepared to assess the solubility of ibuprofen with increasing concentrations of SP. The increase in Soluplus concentration resulted in increased solubility of the ibuprofen in both water and PBS. The highest solubility of the ibuprofen was observed in PBS at 8% Soluplus. The effect of different concentrations of Soluplus on enhancing the solubility of ibuprofen in water and PBS (7.4) at 37 °C is shown in Figure 4.

The amount of drug soluble in water and PBS without Soluplus was 18.7 and 58.8 µg/mL, respectively, which increased to 258.9 and 489.6 µg/mL with 8% of SP. The solubility of ibuprofen in both water and PBS in the presence of SP was significantly high (*p* = 0.001). Several studies have previously reported the use of SP as a solubility enhancer of BCS class II drugs due to its potential for micellization [22,23]. The results obtained in our study were in agreement with previously established solubility enhancement properties of SP [24]. The amphiphilic nature of SP enables the process of micellization to take place in aqueous solutions above its CMC value; this was achieved at a concentration of 0.08 g/mL of SP in PBS 7.4, which justifies the enhanced solubility of ibuprofen achieved in aqueous medium with SP [22]. A lipophilic compound can be incorporated in the core of micelles in an aqueous solution; it is assumed that the lipophilic portion of the forming polymer is included in the core and the polar portion forms the shell; thus, the localization of an ibuprofen molecule inside a micelle is dependent on its degree of lipophilicity, and ibuprofen is a highly lipophilic drug, making it an ideal candidate for micellization with SP [22].

### 2.4. Fabrication of Drug-Loaded Microneedles

**Figure 5:**
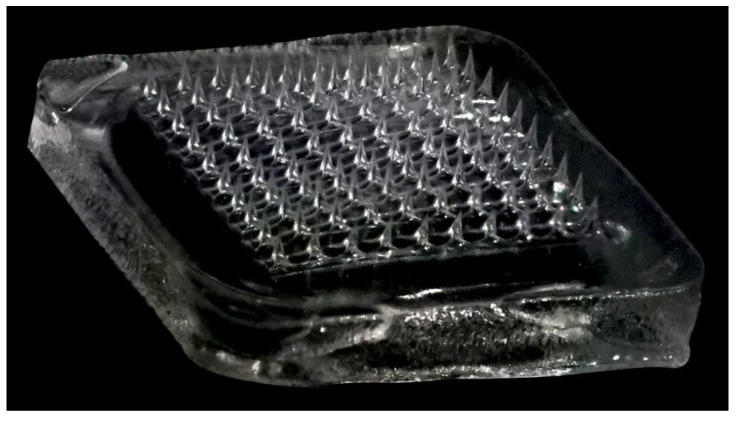
Fabricated MN array patch of ibuprofen.

The PDMS micro-molds were used to fabricate dissolving MNs by the solvent casting method. Different concentrations of PVA solutions (15%, 20%, 22.5%, and 25% *w*/*v*) were used for the fabrication process and analyzed for physical characterization and in vitro drug release. Depending on the physical properties and drug release profile, 25% *w*/*v* PVA was selected as the matrix-forming material to incorporate the drug. The polymer-drug solution was mixed *w*/*w* in a ratio of 1:1. Figure 5 is a representative photograph of the dissolving MN array.

The MNs were fabricated by vacuum filling the molds; a drug-free backing layer of the polymer formed the baseplate to hold the microneedle projections. The process of casting and fabrication was performed at room temperature by applying vacuum for 10 min to yield a 10 × 10 array comprising 100 individual MN projections per array, each with a height of 800 μm, a base width of 200 μm, and an interspacing of 500 μm on an array area of approximately 8 mm × 8 mm. The dimensions of the needles were confirmed with SEM.

### 2.5. Scanning Electron Microscopy (SEM)

**Figure 6:**
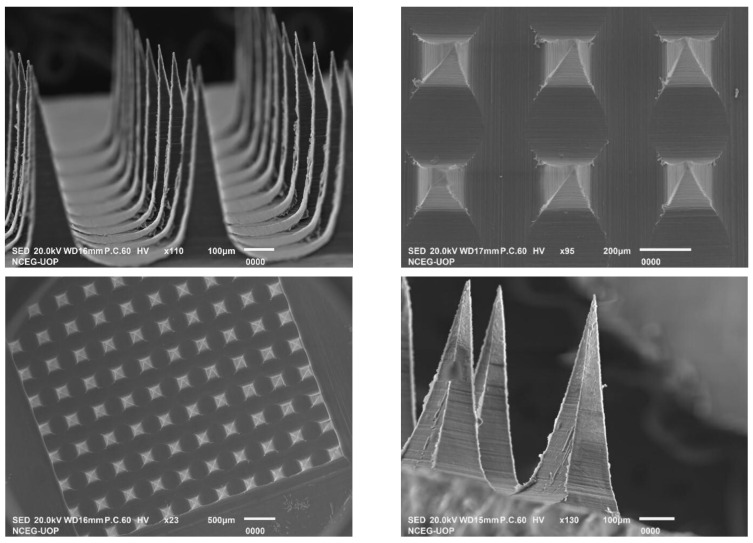
SEM images of the fabricated microneedle patch.

SEM studies (Figure 6) revealed that the microneedle patch has a baseplate with 100 microneedle projections that are evenly distributed. As evident from the SEM image, the formed needles were of pyramid shape with a four-cornered base and a facet width of 200 µm that narrowed to a fine tip over a length of 800 µm. This was consistent with the standard sizes of the PDMS molds provided by Micropoint Technologies.

### 2.6. Mechanical Strength/Breaking Strength

The MN array must have enough mechanical strength to penetrate the skin without mechanical failure. The breaking strength of prepared dissolving MN patches was assessed using a universal testing machine (UTM) in compression mode, which continuously recorded the compression force against the deflection required for microneedle breakage (n = 3). The breaking strength of both placebo and drug-loaded MNs was measured. On application of a set force of 35 N, none of the needles fractured. The results obtained are in consonance with the previous studies [25].

**Figure 7:**
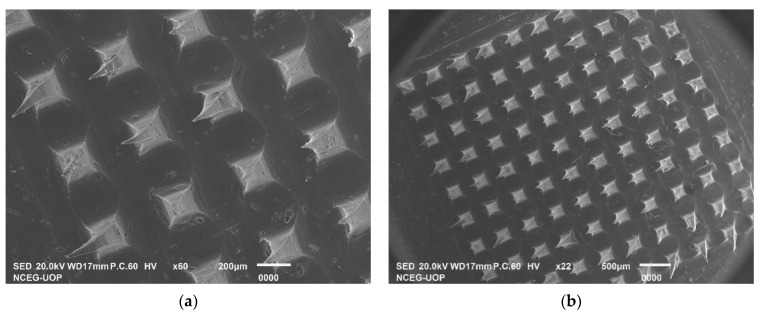
SEM Images of MN patch after applying a set force of 35 N show deformed tips (**a**) and (**b**) showing different magnifications of the array.

The microneedle insertion force into the skin barrier is approximately 0.098 N/needle [26], hence the prepared MN patch had enough strength to effectively penetrate the skin’s stratum corneum. For microneedles to perform effectively, it is essential that they withstand a force that does not cause them to break easily [27,28]. A force of 350 N was applied to the MN patch; the needles were deformed at a force of 3.5 N/needle but did not break; this suggests that the microneedle patch was able to withstand significant force before reaching its breaking point. This was due to the good physical strength of PVA, as previously reported [29], and also to the high percent incorporation of the drug, which imparts additional strength to the formed MNs. It is considered that the addition of SP also imparted mechanical strength to the MNs based on previous studies reporting the impact of surfactants on the performance of MNs [16]. Figure 7a,b represents the SEM images of the MN patch after mechanical strength testing.

### 2.7. Insertion Studies

MN arrays were inserted using a spring applicator into eight-layer folded PF sheets as a model for measuring skin insertion [27,28]. Parafilm has been previously used in many studies to assess the insertion/mechanical properties of microneedles [30]. The MN array patch was inserted into the folded sheets and held for 30 s (n = 3); after removal, the sheets were observed for the total number of perforations created. The percent insertion of MNs in each layer was calculated using Equation (1). The thickness of a single parafilm was 130 µm; the eight-layer folded sheets presented a thickness of 1040 µm. All the needles had penetrated up to the third layer of parafilm, indicating 100% insertion to 390 µm depth. The MNs were able to insert 80% into the fourth layer.

**Figure 8:**
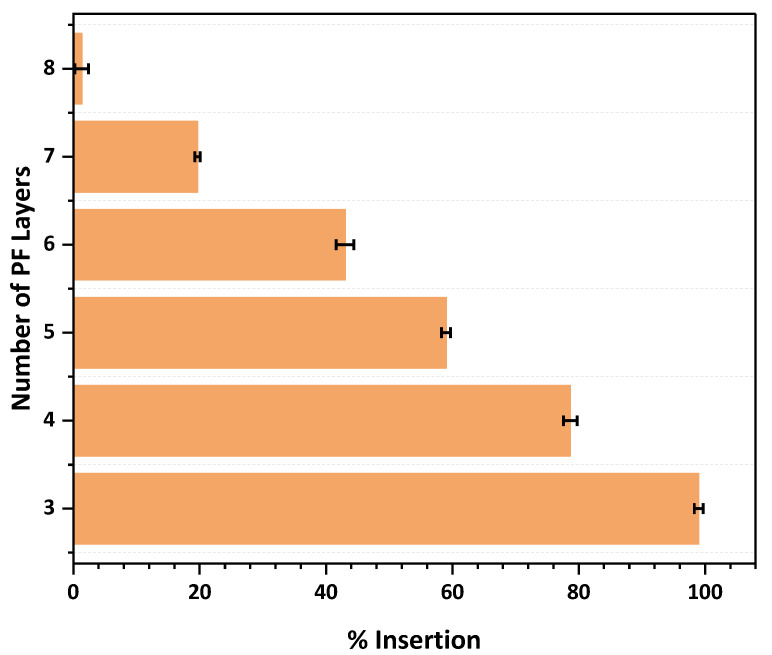
% Insertion of microneedle array across PF sheets.

MNs must pierce the human stratum corneum (10 to 20 μm) to serve as a substitute for common hypodermic needles, which is easily achieved by the MN array with a needle height of 800 μm; rather, the MNs go deep down to the dermis, approximately till 500 μm. This is consistent with a previous study [31]. Figure 8 shows the percent insertion of the array starting from the topmost first layer until the last eighth layer.

### 2.8. Drug Content

The drug content was calculated as the percentage recovery of ibuprofen from MN array patches in triplicates (n = 3) by using the formula given as Equation 2 with the help of HPLC. The calibration curve was obtained for ibuprofen standard solution and plasma for different concentrations of ibuprofen ranging from 0.5 to 5 µg/mL; the R^2^ values were 0.9998 and 0.9994 for standard and spiked plasma, respectively. The recovery was 100% ± 0.67. Each MN array patch contained a total of approximately 55 µg of the drug.

### 2.9. In Vitro Drug Release Profile

**Figure 9:**
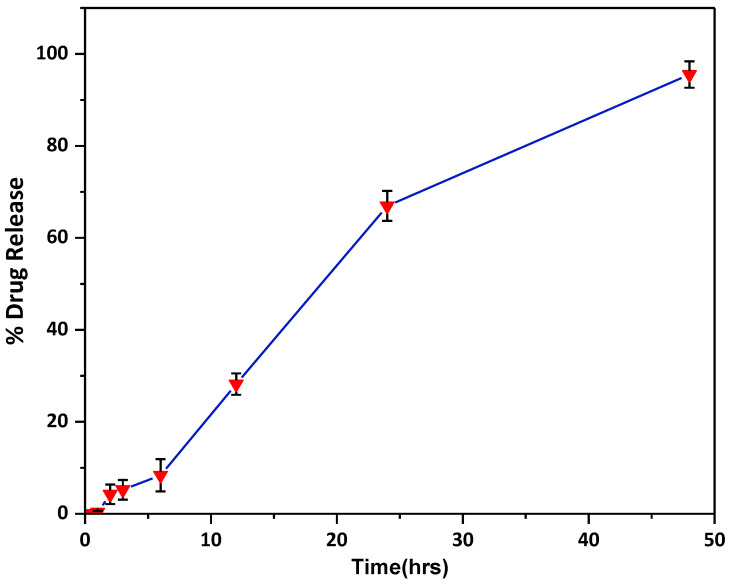
Cumulative drug release profile of ibuprofen microneedles.

The cumulative drug release of ibuprofen from the MN patch (n = 3) is shown in Figure 9.

It suggested that the prepared MNs were able to release the drug in a controlled and predicted manner over a period of 48 h; the results displayed initial burst release, which was followed by sustained release of the drug, confirming a biphasic release pattern. The zero-order kinetic model best fitted the release data (R^2^ = 0.98) as evident in Table 1. The drug release from the MNs was through dissolution, as predicted by the Hixon–Crowell model (R^2^ = 0.98). The n value (1.8) obtained by fitting the Korsemyer and Peppas model acknowledged super-case II transport drug release [32,33]. The super-case II transport corresponds to zero-order kinetics [34] (time-independent release), which is related to the relaxation behavior of the polymer happening upon water permeation into the MNs system as the rate-controlling step [35]. The permeated water results in swelling of the matrix, which in turn causes the degradation and dissolution of the MNs.

### 2.10. In Vivo Release Studies

**Figure 10:**
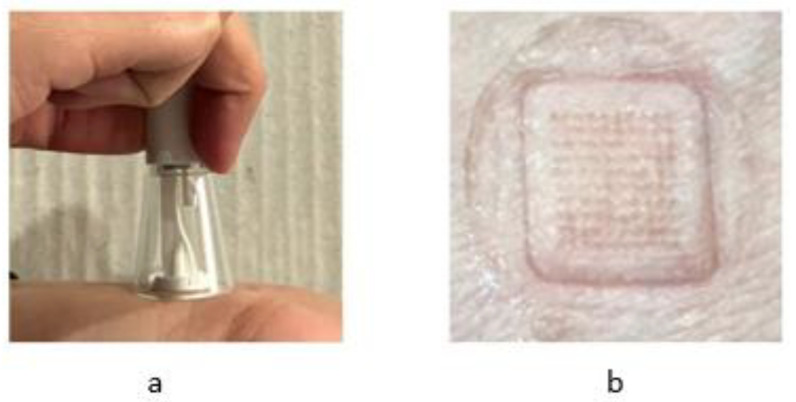
(**a**) Insertion of MN patch and (**b**) skin image after patch removal.

Comparative in vivo non-compartmental analysis (NCA) was carried out to estimate the pharmacokinetic parameters of the marketed oral formulation (200 mg tablet), the topical formulation (10% cream) of ibuprofen, and the fabricated dissolving MN patches. Three MN arrays were applied with the help of a spring applicator and taped with medical sticking to hold them in place for a period of 48 h, as shown in Figure 10.

Three dissolving MN patches were applied to the volar part of the wrist of all the volunteers (n = 18) in the study. The average amount of ibuprofen in each dissolving MN patch was approximately 55 µg. The pharmacokinetic parameters were determined for all three groups. The ANOVA results suggested significant differences among all the groups; to analyze the significance between each group, a post-hoc LSD test was performed. The pharmacokinetic parameters and the post-hoc significance values between MN patch vs. cream and tablet are shown in Table 2.

**Figure 11:**
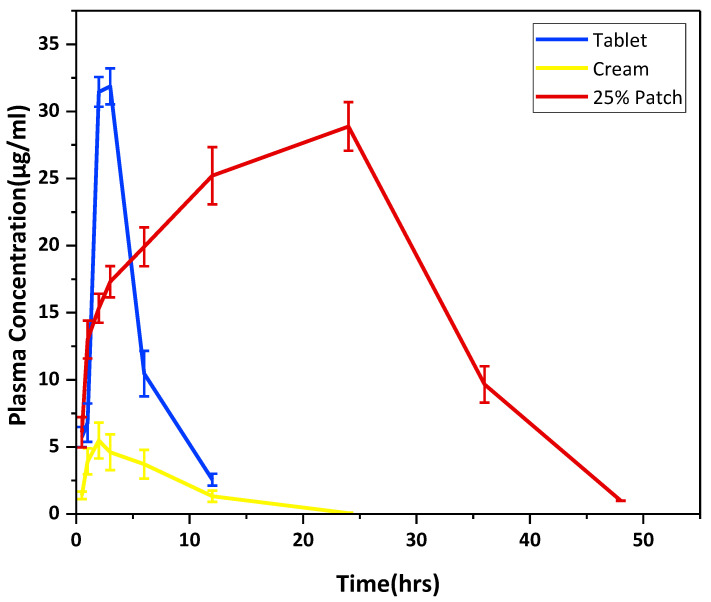
Plasma concentration versus time curve of ibuprofen tablet, ibuprofen cream, and ibuprofen MN patch.

The plasma concentrations vs. time curve shown in Figure 11 indicated that the MN formulation can maintain the drug plasma concentration up to 48 h as compared to tablets and cream.

The t_1/2_, C_max_, T_max_, MRT, AUC, and Cl of prepared microneedles were significantly different from those of tablets and cream, as shown in Table 2. The t_1/2_ of MNs was significantly higher than that of tablets and cream. The higher half-life (*p* = 0.009 and 0.013), as shown in Table 2, is due to the low clearance of ibuprofen from the plasma with continuous and sustained drug release from the dissolving MNs over the studied time period. The *p*-value (0.000) for C_max_ of the prepared MNs was significantly higher than that of the cream; this is due to the fact that the MNs pierced the stratum corneum, thus bypassing the major barriers posed in dermal drug delivery, as in the case of cream, ibuprofen permeates poorly through skin, resulting in low drug absorption and consequently lower plasma drug levels. The C_max_ obtained after administration of MNs was five times higher than that obtained with cream. Despite the low dose of ibuprofen administered through MNs patches (i.e., 165 µg), a comparable C_max_ value was obtained as compared to the tablet formulation (200 mg). This is attributed to the effective skin penetration properties of the microneedles, which deliver the drug deep down to the dermis and achieve optimum bioavailability, whereas the poor gastric absorption serves as a rate-limiting step towards the oral bioavailability of ibuprofen. The first-pass effect also leads to decreased plasma concentrations, as seen with the tablet formulation. The MNs demonstrated a higher bioavailability with an AUC_0-t_ of 831.9 μg/mL*h as compared to tablets and cream, which were 157.84 and 43.90 μg/mL*h, respectively. The AUC of the MNs was 5.3 and 18.95 times higher than the commercial tablet and cream, respectively. The results were statistically significant, as evident from the *p*-value. The MRT observed with MNs was significantly higher (*p* = 0.000) than that observed with the other two formulations. This was due to the slow, sustained release of the drug from the MN patch, which delivered the drug over a longer period of time and had low clearance from the system; hence, the drug resided more in the plasma with a higher MRT value. The slow, controlled release of the drug led to slow clearance, which is significantly lower for MNs than that for tablets and cream. The pharmacokinetic parameters correlate with the in vitro dissolution profile of the MNs.

## 3. Materials and Methods

### 3.1. Materials and Analytical Instruments

Ibuprofen and Naproxen were purchased from Macklin Biochemical Co., Ltd. (Shanghai, China). Polydimethylsiloxane (PDMS) molds containing 100 microneedles with an 800 µm height, 200 µm base, and 500 µm pitch were purchased from Micropoint Technologies Pte, Ltd. (Singapore, Singapore). Poly vinyl alcohol 1500 (Lot: P01380a2) was obtained from Sigma Aldrich, Darmstadt, Germany. Soluplus (Lot: 85937736WO) was obtained from BASF SE (Ludwigshafen, Germany). Acetonitrile (Lot: 1878549) (purity >99.9%) was purchased from Fisher Scientific (Pittsburgh, PA, USA). Methanol (purity >99.9%) (Lot: 1905879) from MERCK, Sigma-Aldrich, Dermastdt, Germany, Dichloromethane (DCM) and potassium dihydrogen phosphate were purchased from Sigma Aldrich (St. Louis, MO, USA). Ortho-phosphoric acid (Lot: K3147213) (Scharlau Chemie, Barcelona, Spain), Sodium Bicarbonate (NaHCO_3_), 99.95% purity (Fluka), Sodium Chloride, Di-potassium hydrogen phosphate (Lot: 60221), Potassium Chloride (Lot: SZBD1740V) (Sigma Aldrich, Darmstadt, Germany), and distilled water (Millipore Ultrapure Water Sys. (Milford, CT, USA)) were also procured.

The Perkin Elmer Series 200 HPLC system (Norwalk, CT, USA) was used, which included an autosampler, vacuum degasser, pump, UV-visible detector, and Peltier column oven. To acquire and evaluate the experimental data, a Perkin–Elmer Total Chrom Workstation Version 6.3.1 connected to the HPLC system via a network chromatography interface (NCI 900) was used. The separation was achieved using a Welchrom^®^ (Welch, Shanghai, China) C8, 5 µm, 4.6 × 150 mm analytical column fitted with a guard cartridge of matching chemistry.

### 3.2. Methodology

#### 3.2.1. Preformulation Studies

##### FTIR

The FTIR spectra for the pure drugs ibuprofen, PVA, Soluplus, and their physical mixtures were obtained using the potassium bromide (KBr) disc technique on a PerkinElmer spectrum BX FTIR (PerkinElmer, Waltham, MA, USA). A spectral range of 4000–400 cm^−1^ at a resolution of 4 cm^−1^ was used for measuring the % transmittance.

##### Thermal Analysis (DSC/TGA)

Thermal analysis (DSC technique) recorded the phase transition behavior and enthalpy changes of ibuprofen, PVA, SP, and their physical mixtures. This was performed using the Simultaneous thermal analyzer STA 8000 (Perkin Elmer, Waltham, MA, USA). The process was carried out from 40 to 600 °C at a heating rate of 10 °C min^−1^, under a flow of nitrogen at a rate of 30 mL/min.

Thermogravimetric analysis (TGA) was performed to assess any weight loss against an increase in temperature by applying a heating rate 10 °C min^–1^ in the temperature range of 40–700 °C under an atmosphere of nitrogen with a flow rate of 40 mL min^–1^. The weight loss was measured to see if there were any changes in the thermal stability of the samples.

#### 3.2.2. Solubilization of Ibuprofen

Ibuprofen is poorly soluble in water and other aqueous solvents. Its enhanced solubility was achieved by using a novel, amphiphilic, highly water-soluble caprolactam-poly vinyl acetate-polyethylene glycol graft copolymer (Soluplus^®^) to encourage micellization. Different concentrations of SP were used with water and PBS to select the optimum concentration of SP to achieve maximum solubility. The concentrations studied were 0%, 1%, 2%, 4%, 6%, and 8% *w*/*v*. Ibuprofen, 25% by weight, was gradually added to each 5 mL Soluplus aqueous dispersion in increments. The dispersions were covered with parafilm M and allowed to stir in a shaking water bath at 36 °C and 200 rpm for 24 h, centrifuged at 13,000 rpm and 10 °C for 1 h to separate the undissolved drug, and the supernatants were diluted with acetonitrile (ACN) and analyzed with HPLC-UV.

#### 3.2.3. Preparation of Casting Material (Drug-Polymer Blend)

Initially, the drug-SP dispersion was prepared with 8% SP in PBS pH 7.4, as described in Section 3.2.2. Following that, a 25% *w*/*v* solution of PVA was prepared in distilled water by dissolving the required amount of PVA in 10 mL of pre-heated (80 °C) distilled water for 4 h, until a clear gel-like solution was obtained. It was then allowed to cool to room temperature. To serve as the microneedle casting material, the PVA gel solution and the drug-SP dispersion were mixed in a ratio of 1:1 *w*/*w*. The resulting blend was mixed over a magnetic stirrer at 150 rpm for 5 min at room temperature, and this was allowed to stand for 24 h to remove any bubbles before incorporation into PDMS molds.

#### 3.2.4. Fabrication of Drug-Loaded Microneedles

**Figure 12:**
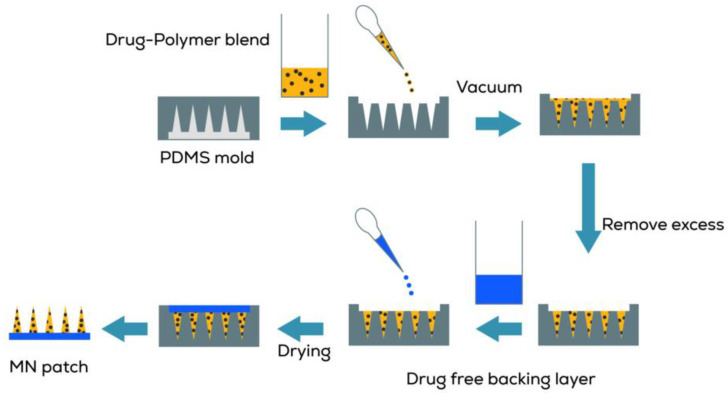
Schematic representation of the fabrication process.

The microneedle patches were prepared by the micromolding technique using a vacuum (−90 kPa) at 25 °C for 10 min. The drug-polymer blend (about 2 µL) was poured on the PDMS mold and subjected to vacuum using an oilless vacuum pump to allow efficient casting of the blend. The excess solution present on the surface of the mold was pipetted off. A drug-free backing layer of the PVA gel solution was poured on the mold and subjected to vacuum again to ensure optimum filling. The molds were allowed to dry at room temperature for 24 h, manually separated from the mold using adhesive tape, and stored in a desiccator until used. Placebo patches were also prepared in the same way. Figure 12 shows the schematic representation of the process.

### 3.3. Characterization of Ibuprofen Microneedles

#### 3.3.1. Scanning Electron Microscopy (SEM)

SEM (JSM-5910, Jeol, Tokyo, Japan) was performed for the morphological characterization and shape of the drug-loaded MN patch. The MN patch was coated with gold and visualized under the Phenom™ SEM system to measure the needle morphology and dimensions.

#### 3.3.2. Mechanical Strength/Breaking Strength

The UTM (universal testing machine) was used to assess the mechanical properties of prepared MN patches in triplicate (n = 3). The drug-loaded MN patch was placed on the flat, rigid aluminum block with the tips of the needles facing upwards. An axial force adjusted perpendicular to the block (i.e., parallel to the MN’s vertical axis) was applied using a stainless-steel cylindrical probe with a flat head of 10 mm diameter. This force pressed against the tips of the MN array at a constant speed of 1.1 mm/s and a set trigger force of 35 N. The UTM recorded the force required to move the metal cylinder as a function of distance, and the deformity in the MN patch was observed through microscopy.

#### 3.3.3. Insertion Test

The skin simulant Parafilm M (PF) was used to evaluate the in vitro skin insertion capability of the prepared MN patches. The 10 × 10 array of MN patches was attached to a spring-operated applicator and then inserted into the stacked layers of PF (n = 3) after a sheet of Parafilm was folded to create an eight-layer film (1 mm thickness) [28]. After the MNs were removed, the punctured film was imaged under the microscope to calculate the percent insertion by the formula given as Equation (1).

(1) $$\mathrm{Percent}\;\mathrm{Insertion}=\frac{\mathrm{Number}\;\mathrm{of}\;\mathrm{holes}\;\mathrm{created}\;\mathrm{in}\;\mathrm{the}\;\mathrm{parafilm}\;\times \;100}{\mathrm{Total}\;\mathrm{no}.\;\mathrm{holes}\;\mathrm{microneedles}}$$

#### 3.3.4. Drug Content

In order to determine the percentage recovery of ibuprofen from dissolving the MN array, a single microneedle patch was soaked in distilled water in a 15 mL Eppendorf tube and subjected to shaking in a heated shaking water bath for 24 h at 80 °C to completely dissolve the polymers PVA and Soluplus (n = 3). It was centrifuged for 15 min at 10 °C and 3000× *g*. The supernatant was discarded, and the precipitate containing ibuprofen was reconstituted with the mobile phase. This solution was sonicated, vortex-mixed, and filtered through a microfilter. The drug content and percentage recovery, as well as the in vitro and in vivo release of ibuprofen, were determined by HPLC. Briefly, a 50 μL sample was injected into the Welchrom^®^ (Welch, Shanghai, China) column (C8, 4.6 150 mm), and the mobile phase was comprised of ACN:Phosphoric acid buffer, pH 2.3 (55:45 *v*/*v*), and was pumped with a flow rate of 1 mLmin^−1^. The UV detection was carried out at 220 nm, using naproxen sodium as an internal standard. The percent drug recovery was calculated by using the formula given in Equation 2.

(2) $$\mathrm{Percent}\;\mathrm{drug}\;\mathrm{recovery}=\frac{\mathrm{Absorbance}\;\mathrm{of}\;\mathrm{the}\;\mathrm{sample}\;\left(\mathrm{MN}\;\mathrm{patch}\right)\times \;100}{\mathrm{Aborbance}\;\mathrm{of}\;\mathrm{standard}\;(\mathrm{Ibu})}$$

#### 3.3.5. In Vitro Drug Release Profile

The drug-loaded microneedle patches in triplicate were assessed for drug release profiles (n = 3). The study was carried out using Franz cells with Parafilm M [36]. Three layers of Parafilm M stacked together were mounted on the Franz diffusion cell. The acceptor compartment was filled with the dissolution medium (phosphate buffer pH 7.4) and stirred at 600 rpm, maintaining the temperature at 37 ± 1 °C by circulating water via a peristaltic pump [15]. A drug-loaded microneedle patch was inserted on the stacked parafilm and secured by clamping between the donor and receptor chambers. An aliquot of 0.5 mL was collected from the sampling arm at predetermined time intervals (0.5, 1, 2, 3, 6, 12, 24, and 48 h.) and replaced with an equal volume of dissolution medium to maintain sink conditions. The drug content at different time intervals was assayed by HPLC method, as discussed.

#### 3.3.6. In Vivo Release Studies

The in vivo study was carried out on healthy human volunteers. The study was approved by the Department of Pharmacy at the University of Peshawar ethical committee (505/EC-FLES-UOP/2022). The volunteers were divided into three groups (6 volunteers each n = 18). Group A was administered a 200 mg ibuprofen tablet, and Group B received an amount equivalent to 200 mg of ibuprofen in a 10% ibuprofen cream. Whereas group C was administered with the fabricated MN arrays containing 165 µg of the drug. Blood samples at predetermined time intervals (0, 0.5, 1, 2, 3, 6, 12, 24, and 48 h) were collected in ethylenediaminetetraacetic acid (EDTA) glass tubes and centrifuged at 1600× *g* for 10 min at 4 °C. Plasma was collected and stored at −20 °C for further analysis. A total amount of 200 µL of plasma was taken in an Eppendorf tube, and 10 µL (1µg/mL) of the internal standard (naproxen sodium) was added to it. The sample was vortex-mixed for 5 min, acidified with 120 µL of 1 M HCl, vortexed, followed by the addition of 500 µL of methanol to effect protein precipitation, and centrifuged at 10 °C for 10 min at 5500 rpm. The supernatant was collected, and the procedure was repeated once. The supernatant was dried under a stream of nitrogen at 40 °C. The dried sample was reconstituted with the mobile phase and analyzed for ibuprofen content with HPLC-UV, as discussed.

## 4. Conclusions

The approach of enhancing the solubility of the ibuprofen using Soluplus as a solubility enhancer and distributing the drug uniformly and evenly in the PVA polymer was successfully achieved by fabricating dissolving MNs with sufficient mechanical strength that were non-brittle and smooth, as evident from SEM images. The MNs delivered the drug in a predictable manner via dissolution of the polymer-drug matrix, as confirmed by the Hixon–Crowell model. The MNs successfully crossed the skin’s stratum corneum barrier, which resulted in active absorption of ibuprofen in plasma as compared to its passive permeation from the cream, which again resulted in higher bioavailability with a much lower dose (165 µg) as compared to tablets and cream (200 mg). It was concluded that the solubility achieved with SP by the direct dissolution method resulted in stable aqueous dispersions of the drug that are effectively incorporated into the fabrication process, overcoming the fragility and breakage of the MN patches that were observed by Donnelly et al. The FTIR study showed that the excipients used were compatible with ibuprofen. The thermal analysis also suggested that the MNs were stable enough, and the Soluplus and the polymer imparted no degradative effect on the thermal stability of ibuprofen. In vitro studies as well as in vivo pharmacokinetic evaluations confirmed the potential of MNs to provide sustained delivery of ibuprofen through the transdermal route. The high bioavailability and sustained release with lower doses will not only reduce the side effects but also reduce the cost of therapy and patient compliance.

## Figures and Tables

**Table 1:** R^2^ and k values obtained by fitting different kinetic models.

Model	R^2^	K	n	Regression Equation (y=)
Zero order	0.98	2.220	--	2.2055x−2.2204
First order	0.572	0.280	--	0.0606x−0.2803
Hixson and Crowell	0.98	102.2	--	−2.2055x + 102.22
Higuchi	0.95	22.5	--	16.6x−22.5
Korsemeyer and Peppas	0.947	--	1.885	1.8853x−0.82

**Table 2:** Pharmacokinetic parameters for ibuprofen tablet, cream, and fabricated MN patch after NCA.

Parameter	Unit	Tablet	Cream	Ibu 25% MNs	Sig. (Post HOC Analysis)	Sig. (Post HOC Analysis)
Mean ± SD	Mean ± SD	Mean ± SD	MN- Cream	MN-Tab		
t_1/2_	Hr	2.48 ± 0.33	2.29 ± 0.16	5.2 ± 0.1	* 0.009	* 0.013
T_max_	Hr	2.66 ± 0.41	2.33 ± 0.41	24.0 ± 0.0	* 0.000	* 0.000
C_max_	μg/ml	32.66 ± 1.73	5.73 ± 0.24	28.7 ± 0.5	* 0.000	* 0.078
AUC_0-t_	μg/mL·h	157.84 ± 0.83	43.90 ± 2.44	831.9 ± 30.8	* 0.000	* 0.000
AUC_0–∞_	μg/mL·h	167.05 ± 4.72	43.95 ± 2.45	841.0 ± 31.1	* 0.000	* 0.000
AUMC_0–∞_	μg/mL·h^2^	780.63 ± 90.88	259.23 ± 7.73	16,387.0 ± 463.3	* 0.000	* 0.000
MRT_0–∞_	H	4.66 ± 0.42	5.91 ± 0.19	19.5 ± 0.3	* 0.000	* 0.000
Cl/F	(mg)/(μg/mL)/h	4.94 ± 0.14	18.85 ± 1.09	1.0 ± 0.0	* 0.000	* 0.000

* = The values are statistically significant (*p* < 0.05).

## Data Availability

Data is contained within the article.
